# Fluoride exposure and sleep patterns among older adolescents in the United States: a cross-sectional study of NHANES 2015–2016

**DOI:** 10.1186/s12940-019-0546-7

**Published:** 2019-12-09

**Authors:** Ashley J. Malin, Sonali Bose, Stefanie A. Busgang, Chris Gennings, Michael Thorpy, Robert O. Wright, Rosalind J. Wright, Manish Arora

**Affiliations:** 1 Department of Environmental Medicine and Public Health, Icahn School of Medicine at Mount Sinai, New York, NY USA; 2 Department of Pediatrics, Icahn School of Medicine at Mount Sinai, New York, NY USA; 3 Division of Pulmonary, Critical Care and Sleep Medicine, Icahn School of Medicine at Mount Sinai, New York, NY USA; 4 The Saul R. Korey Department of Neurology, Albert Einstein College of Medicine, New York, NY USA

**Keywords:** Fluoride, Sleep, Sleep apnea, Adolescents, United States

## Abstract

**Background:**

Fluoride from environmental sources accumulates preferentially in the pineal gland which produces melatonin, the hormone that regulates the sleep-wake cycle. However, the effects of fluoride on sleep regulation remain unknown. This population-based study examined whether chronic low-level fluoride exposure is associated with sleep patterns and daytime sleepiness among older adolescents in the United States (US).

**Method:**

This cross-sectional study utilized data from the National Health and Nutrition Examination Survey (2015–2016). We analyzed data from adolescents who had plasma fluoride (*n* = 473) and water fluoride (*n* = 419) measures and were not prescribed medication for sleep disorders. Relationships between fluoride exposure and self-reported sleep patterns or daytime sleepiness were examined using survey-weighted linear, binomial logistic or multinomial logistic regression after covariate adjustment. A Holm-Bonferroni correction accounted for multiple comparisons.

**Results:**

The average age of adolescents was 17 years (range = 16–19). Median (IQR) water and plasma fluoride concentrations were 0.27 (0.52) mg/L and 0.29 (0.19) μmol/L respectively. An IQR increase in water fluoride was associated with 1.97 times higher odds of reporting symptoms suggestive of sleep apnea (95% CI: 1.27, 3.05; *p* = 0.02), a 24 min later bedtime (B = 0.40, 95% CI: 0.10, 0.70; *p* = 0.05), a 26 min later morning wake time (B = 0.43, 95% CI: 0.13, 0.73; *p* = 0.04), and among males, a 38% reduction in the odds of reporting snoring (95% CI: 0.45, 0.87, *p* = 0.03).

**Conclusions:**

Fluoride exposure may contribute to changes in sleep cycle regulation and sleep behaviors among older adolescents in the US. Additional prospective studies are warranted to examine the effects of fluoride on sleep patterns and determine critical windows of vulnerability for potential effects.

## Background

Fluoride is an ion that occurs naturally in the earth’s crust and can also be released through industrial processes [1–3]. In the United States (US), approximately 74% of the population on public water supplies receives chemically fluoridated water for tooth decay prevention [4]. Many benefits of fluoride have been reported for oral health and it is regarded as a major public health achievement [5]. Fluoride strengthens teeth in part by replacing the hydroxyl ions in enamel hydroxyapatite to form fluor-hydroxyapatite or fluorapatite which are more crystalized structures [6]. Recently, there has been interest in understanding the adverse effects of fluoride exposure to balance the benefits with any potential risks. For example, excess fluoride exposure during tooth formation can interfere with enamel mineralization and cause dental fluorosis – visually detectable changes in tooth enamel ranging from mild to severe [7, 8]. Moreover, fluoride from chronic systemic exposure accumulates highly in other bodily tissues that contain hydroxyapatite, such as the pineal gland [9–11], which may increase the risk of toxicity in these areas.

The pineal gland is a small pea-shaped gland located between the two cerebral hemispheres and outside of the blood-brain barrier. It is comprised of both soft tissue and hydroxyapatite crystals. Its primary function is to synthesize melatonin, an antioxidant that plays an essential role in maintenance of normal sleep patterns [12]. Fluoride accumulation in pineal gland hydroxyapatite is present in higher concentrations than in any other part of the body, including bones and teeth [9, 10]. In 2006, a National Research Council report concluded that fluoride is likely to affect pineal gland function and cause decreased melatonin production which could contribute to a variety of effects in humans [13]. However, to our knowledge, no published studies have examined effects of fluoride exposure on melatonin production or sleep regulation in either humans or animals.

Therefore, our study aimed to examine whether fluoride exposure (measured in blood plasma and household tap water) is associated with sleep patterns among older adolescents in the US. We hypothesized that greater fluoride exposure would be associated with changes in sleep patterns and more frequent daytime sleepiness.

## Methods

### Participants

This study utilized data from the National Health and Nutrition Examination Survey (NHANES) collected from 2015 to 2016 because this cycle included both fluoride biomonitoring data and a breadth of self-reported sleep outcome measures. Plasma fluoride concentrations were measured among 2145 participants aged 6–19 years and household tap water fluoride concentrations were measured among 3987 participants aged 0–19 years. Sleep outcomes were assessed among participants aged 16 and over, and thus we limited our analyses to participants ages 16–19. We included participants who had either plasma or water fluoride measurements, complete covariate data, and data on at least one sleep outcome measure. We excluded 3 participants who reported taking medications for sleep disorders. There were 512 participants who met inclusion criteria. Of those, 473 had plasma fluoride measurements and were included in analyses. For analyses including water fluoride as a predictor, there were 503 participants who met inclusion criteria and we excluded an additional 84 who reported that they did not drink tap water, resulting in a final analytic sample of 419. Participant selection is depicted in Additional file 1: Figure S1. A comparison of demographic characteristics between the current study sample and all adolescents aged 16–19 in NHANES is presented in Additional file 2: Table S1. Sampling weights were applied to account for the complex NHANES survey design as recommended by the National Center for Health Statistics (NCHS). The weighted samples for plasma and water fluoride analyses represented 12,531,822 and 11,577,700 U.S. adolescents, respectively.

### Fluoride measures

Fluoride concentrations were measured in blood plasma and household tap water. Collection times of blood and tap water were not standardized. Plasma fluoride concentrations were measured using an ion-specific electrode and hexamethyldisiloxane method [14], while household tap water samples were measured electrometrically with an ion-specific electrode [15]. Plasma and water samples were measured for fluoride in duplicate (using the same sample) and the average of the two values was publicly released. Fluoride measurements were conducted at the College of Dental Medicine, Georgia Regents University, Augusta, GA. The lower limit of detection (LLOD) for plasma fluoride was 0.25 nmol, and the LLOD for water fluoride was 0.10 mg/L. Approximately 86 and 100% (all) of participants had values above the LLOD for water fluoride and plasma fluoride respectively. Laboratory generated values for water fluoride concentrations below the LLOD were utilized in analyses; however, values below the LLOD were imputed as $LLOD/\sqrt{2}$ for descriptive statistics.

### Sleep measures

Sleep habits and sleep disorders were ascertained through questionnaires in participants’ homes by trained staff using the Computer-Assisted Personal Interview (CAPI) system. The CAPI system is equipped with built-in consistency verification to reduce data entry errors [16]. The questions included in the sleep questionnaire were not validated.

#### Bedtime/wake time and sleep duration

Participants were asked to estimate the time that they usually get in and out of bed on weekdays or workdays, excluding naps (i.e. *What time do you/does study participant (SP) usually go to sleep on weekdays or workdays? What time do you/does SP usually wake up on weekdays or workdays?).* Using bedtime and wake time data, the number of hours of sleep per night (i.e. nighttime sleep duration) was calculated by the NCHS.

#### Sleep disturbances

Participants were asked how often they snore (i.e. *In the past 12 months, how often did you/SP snore while you were/s/he was sleep*?), and how often they experience symptoms suggestive of sleep apnea (i.e. “*In the past 12 months, how often did (you/SP) snort, gasp, or stop breathing while (you were/s/he was) asleep?*)*.* Responses to these questions were categorized as “never”, “rarely – 1-2 nights a week”, “occasionally - 3-4 nights a week”, or “frequently - 5 or more nights a week”. When participants implied that they did not know whether they exhibit these behaviors, the interviewer asked whether anyone told them that they do. Participants were also asked directly whether they experience sleep disturbances (i.e. “*Have you/Has SP ever told a doctor or other health professional that you have/s/he has trouble sleeping?”)*. Responses were dichotomized as “yes” or “no”.

#### Daytime sleepiness

To assess daytime sleepiness, participants were asked *“In the past month, how often did (you/SP) feel excessively or overly sleepy during the day?”*. Responses to this variable were categorized as “never”, “rarely - 1 time a month”, “sometimes - 2-4 times a month”, “Often- 5-15 times a month”, or “Almost always - 16-30 times a month”.

### Covariates

We selected covariates a priori that are empirically associated with fluoride exposure and sleep in existing literature [17–23], including age, sex, body mass index (BMI), race/ethnicity, and the ratio of family income to poverty. The NCHS calculated the ratio of family income to poverty by dividing annual family income by the poverty guidelines for a given survey year.

### Statistical analyses

We applied survey weights from the mobile exam center visit (i.e. MEC weights) for all analyses to account for the clustered sample design, over-sampling, post-stratification, survey non-response, and sampling error. Survey weights also permit generalization to the entire US population [24]. Given that we utilized a dietary variable as exclusion criteria (i.e. tap water consumption) for analyses including water fluoride, we applied reweighted MEC weights to our dietary sample prior to regression analyses with water fluoride, according to NCHS guidance (as describe elsewhere [25]). Results of regression analyses did not change regardless of whether MEC weights or reweighted MEC weights were applied. Means and proportions were calculated for descriptive analyses of demographic variables as well as fluoride exposure and sleep outcome measures. A Pearson correlation examined the relationship between logarithm (base 2)-transformed plasma and water fluoride concentrations. To examine the relationship between fluoride exposure and sleep duration, sleep duration was transformed into a 3-category variable in which short duration = 1, normal duration = 2 and long duration = 3. Normal duration was determined based on sleep duration recommendations by the National Sleep Foundation. 8–10 h is considered normal duration for 16–17-year-olds, and 7–9 h is considered normal duration for 18–19-year-olds [26]. Values below or above this duration were categorized as short or long duration respectively. Survey-weighted multinomial logistic regression were utilized to model sleep duration or daytime sleepiness as a function of plasma or water fluoride concentrations while adjusting for covariates. For regression analyses of fluoride exposure and symptoms suggestive of sleep apnea or snoring, we created dichotomous variables of 0 = never occurs or 1 = occurs 1 or more times per week (i.e. “never” versus “ever”). We utilized this classification to account for potential underestimation of the occurrence of these behaviors and because occurrence of symptoms suggestive of sleep apnea even once per week may indicate underlying sleep dysfunction. We conducted survey-weighted binomial logistic regression to model snoring, symptoms suggestive of sleep apnea or self-reported trouble sleeping as a function of plasma or water fluoride concentrations while adjusting for covariates. Bedtime and wake time variables were converted to numeric format, and the bedtime variable was rescaled prior to analysis such that 7 pm (i.e. the earliest bedtime in our sample) was set at 0 and 7 am (i.e. the latest bedtime in our sample) was set at 12. This allowed us to consider times occurring after midnight as later than times occurring prior to midnight. Associations between fluoride exposure and sleep and wake time were explored using survey-weighted linear regression adjusted for covariates. We explored potentially influential values using a Cook’s Distance estimate and did not identify any. Assumptions of linear and logistic regression were satisfied for all models except that we detected heteroscedasticity in models of fluoride exposure and bedtime/wake time. As such, we conducted unweighted quasi-likelihood estimation models (see Additional file 2: Table S2) to account for this, but results did not appreciably differ from weighted linear regression. Therefore, we present weighted linear regression models herein. No issues with multicollinearity were detected. We included a fluoride*sex interaction term in our models to test for sex-specific associations; however, this term was removed if non-significant. Additionally, we conducted a sensitivity analysis to examine whether adjusting for serum cotinine - a biomarker of nicotine exposure, influenced associations between plasma fluoride concentrations and sleep outcomes. A two-tailed alpha of 0.05 was the criteria for statistical significance for all main effects in regression analyses, while a two-tailed alpha of 0.1 was the criteria for statistical significance for interactions. We applied a Holm-Bonferroni correction to account for multiple comparisons for each fluoride variable whereby each class of sleep outcomes (e.g. sleepiness; sleep duration) was considered a separate test. SAS (V.9.4) software was used for all analyses.

## Results

Descriptive statistics for demographic characteristics, fluoride variables, and sleep outcome measures are presented in Tables 1, 2 and 3 respectively. The average age of participants was approximately 17 years. Household tap water fluoride concentrations generally fell below the Public Health Service recommended concentration of 0.7 mg/L [27]. Specifically, median to 75th percentile water fluoride levels ranged from 0.27 to 0.64 mg/L and the interquartile range (IQR) was 0.52 mg/L. The median (IQR) for plasma fluoride was 0.29 (0.19) μmol/L. Average sleep and wake times were 11 pm and 7 am respectively. Most participants did not report sleep disturbances; however, the majority (83%) of adolescents reported feeling overly sleepy 1–15 times per month as compared to never (10%). Tap water fluoride concentrations and plasma fluoride concentrations were moderately positively correlated (*r* = 0.41, *p* < 0.0001).

**Table 1:** Demographic characteristics according to study sample

	Overall sample	Plasma fluoride sample	Water fluoride sample^a^
	*n* = 512 *N* = 13,503,522	*n* = 473 *N* = 12,531,822	*n* = 419 *N* = 11,577,700
Age; Mean (SE)	17.24 (0.03)	17.26 (0.04)	17.22 (0.04)
Ratio family income to poverty; Mean (SE)	2.55 (0.18)	2.52 (0.18)	2.64 (0.19)
Sex; N (%)			
Male	6,627,073 (49.08)	6,210,909 (49.56)	5,810,794 (50.19)
Female	6,876,449 (50.92)	6,320,912 (50.44)	5,766,905 (49.81)
Race/ethnicity; N (%)			
Mexican American	2,142,458 (15.87)	2,117,863 (16.90)	1,701,676 (14.70)
Other Hispanic	880,521 (6.52)	859,924 (6.86)	632,563 (5.46)
Non-Hispanic White	7,387,374 (54.71)	6,800,039 (54.26)	6,696,365 (57.84)
Non-Hispanic Black	1,734,001 (12.84)	1,491,071 (11.90)	1,449,755 (12.52)
Non-Hispanic Asian	702,796 (5.20)	639,594 (5.10)	553,402 (4.78)
Other Race, including Multi-Racial	656,373 (4.86)	623,330 (4.97)	543,938 (4.70)
BMI categories; N (%)^b^			
Underweight	529,432 (3.94)	515,275 (4.14)	470,519 (4.09)
Normal weight	7,428,420 (55.32)	6,734,756 (54.07)	6,351,579 (55.15)
Overweight	2,780,123 (20.70)	2,562,271 (20.57)	2,458,759 (21.35)
Obese	2,690,339 (20.03)	2,644,312 (21.23)	2,235,201 (19.41)

*Note.* Sampling weights were applied for calculation of demographic descriptive statistics and therefore Ns for frequencies represent the *weighted* sample size. Re-weighting for the water fluoride sample was not applied for the calculation of descriptive statistics above.^a^ Participants who reported that they did not drink the tap water were excluded; ^b^ *n* = 507 for entire sample, *n* = 468 for plasma F sample and *n* = 415 for water F subsample due to missing data for this variable

**Table 2:** Descriptive statistics for fluoride exposure measures

Measure	Arithmetic mean	Standard error	Median	5th percentile	95th percentile
Plasma fluoride (μmol/L) ^a^	0.35	0.02	0.29	0.14	0.67
Tap water fluoride (mg/L)^b^	0.39	0.05	0.27	0.07	0.81

*Note.* Sampling weights were applied for calculation of all descriptive statistics. For water fluoride, descriptive statistics remained the same following reweighting of sampling weights; ^a^*n* = 473 (weighted *N* = 12,531,822); ^b^*n* = 419 (weighted *N* = 11,577,700)

**Table 3:** Descriptive statistics for sleep outcome measures according to study sample

	Overall *n* = 512 *N* = 13,503,522	Plasma fluoride sample *n* = 473 *N* = 12,531,822	Water fluoride sample *n* = 419 *N* = 11,577,700
Sleep duration^a^; N (%)			
Less than recommended	3,912,903 (29.08)	3,616,486 (28.97)	3,519,194 (30.52)
Recommended	7,763,278 (57.70)	7,181,883 (57.53)	6,549,336 (56.80)
More than recommended	1,779,421 (13.22)	1,685,533 (13.50)	1461, 250 (12.67)
Sleep apnea symptoms; N(%)^b^			
Never	11,968,414 (89.81)	11,010,121 (89.11)	10,217,865 (89.51)
At least 1 night per week	1,358,534 (10.19)	1,345,127 (10.89)	1,196,827 (10.49)
Snoring; N(%)^c^			
Never	7,564,062 (58.07)	6,919,753 (57.40)	6,602,728 (59.35)
At least 1 night per week	5,462,602 (41.93)	5,135,211 (42.60)	4,521,444 (40.65)
Trouble sleeping; N(%)			
Yes	1,874,575 (13.88)	1,764,264 (14.08)	1,629,889 (14.08)
No	11,628,947 (86.12)	10,767,558 (85.92)	9,947,810 (85.92)
Daytime sleepiness			
Never	1,307,017 (9.68)	1,185,750 (9.46)	1,120,797 (9.68)
Rarely – 1 time a month	2,789,385 (20.66)	2,532,161 (20.21)	2,321,968 (20.06)
Sometimes – 2-4 times a month	4,255,661 (31.52)	4,047,114 (32.29)	3,638,287 (31.43)
Often – 5-15 times a month	4,104,292 (30.39)	3,912,334 (31.22)	3,596,810 (31.07)
Almost always – 16-30 times a month	1,047,168 (7.75)	854,464 (6.82)	899,838 (7.77)
Wake time; Mean (SE)^a^	7.15 (0.09)	7.17 (0.10)	7.15 (0.09)
Bedtime; Mean (SE)^a^	4.00 (0.10)^d^	4.02 (0.11)	4.02 (0.11)

*Note.* ^a^ There was one missing participant for this outcome; ^b^ Overall sample *n* = 505, plasma fluoride sample *n* = 466, and water fluoride sample *n* = 413 due to missing data on this variable. ^c^ Overall sample *n* = 493, plasma fluoride sample *n* = 454, and water fluoride sample *n* = 402 due to missing data on this variable

^d^Bedtime was rescaled such that the earliest bedtime (i.e. 7 pm) = 0 and the latest bedtime (i.e. 7 am) = 12*;* bedtime of 11 pm = 4; wake time was not rescaled and therefore the numeric time is presented. For example, 7.15 is equal to 7:09 am (i.e. the 7th hour and 0.15 of 60 min). Sampling weights were applied for calculation of all descriptive statistics. For water fluoride, mean (SE) and percentages remained the same following reweighting of sampling weights; therefore, frequencies of the sample prior to re-weighting are reported

Regression results for water fluoride and sleep outcomes are presented in Table 4**.** Binary logistic regression adjusted for covariates showed that higher water fluoride concentrations were associated with higher odds of reporting symptoms suggestive of sleep apnea (OR = 1.97, 95% CI: 1.27, 3.05, *p* = 0.02). Specifically, each IQR (i.e. 0.52 mg/L) increase in water fluoride was associated with 1.97 times greater odds of self-reported *snorting, gasping, or stopping breathing* while asleep *ever* as compared to *never.*

**Table 4:** Associations between water fluoride and sleep measures

Outcomes	N	Estimates (95% CI)	Uncorrected *p*	Holm-Bonferroni corrected *p*
Sleep duration	418		0.487^†^	
Less than recommended		1.35 (0.83,2.22)	0.23	0.46
Recommended (ref)		–	–	–
More than recommended		1.10 (0.58, 2.11)	0.76	1.00
Sleep apnea symptoms	413			
Never (ref)		–		
At least once per week		1.97 (1.27, 3.05)	0.002	0.02*
Snoring	402			
Never (ref)		---		
At least once per week		0.62 (0.45, 0.87)^a^	0.005	0.03*
Daytime sleepiness	419		0.220^ǂ^	
Never (ref)		–		
Rarely		1.91 (1.08, 3.38)	0.03	0.08
Sometime		1.50 (0.88, 2.57)	0.14	0.41
Often		2.06 (1.09, 3.89)	0.03	0.08
Almost always		1.53 (0.86, 2.74)	0.15	0.46
Trouble sleeping	419			
No (ref)		–		
Yes		1.02 (0.64, 1.62)	0.93	0.93
Bedtime	418	0.40 (0.10, 0.70)	0.01	0.05*
Wake time	418	0.43 (0.13, 0.73)	0.008	0.04*

*Note.* All estimates are odds radios (ORs) except for bedtime and wake time which are unstandardized Beta estimates; ORs and Beta estimates reflect the change in outcome for each IQR (i.e. 0.52 mg/L) increase in water fluoride concentration. Regression analyses were adjusted for age, sex, race/ethnicity, body mass index, and ratio of family income to poverty. Reweighted MEC weights were applied to these regression analyses; *Significant at *p* ≤ 0.05 after Holm-Bonferroni correction. †The *p*-value for a Type 3 Analysis of Effects with 2 degrees of freedom; ǂ The *p*-value for a Type 3 Analysis of Effects with 4 degrees of freedom; ^a.^ Odds ratio for association between water fluoride and snoring among males; interaction between sex and water fluoride in predicting snoring (B = 1.35, *p* < 0.001)

Linear regression adjusted for covariates showed that higher water fluoride concentrations were also associated with later bedtime and wake time = (B = 0.40, 95% CI: 0.10, 0.70, *p* = 0.05 for bedtime and B = 0.43, 95% CI: 0.13, 0.73, *p* = 0.04 for wake time). Specifically, each IQR increase in water fluoride was associated with going to bed approximately 24 min later and waking up approximately 26 min later.

Higher water fluoride concentrations were associated with higher odds of reporting daytime sleepiness “rarely” or “often” as compared to “never”; however, these associations became borderline statistically significant after the holm-bonferroni correction was applied (OR = 1.91, 95% CI: 1.08, 3.38, *p* = 0.08 and OR = 2.06, 95% CI: 1.09, 3.89, *p* = 0.08 respectively) and the overall Type 3 effect of the analysis (i.e. omnibus test) was not significant (Table 4).

### Evaluation of sex-specific effects

In survey-weighted binary logistic regression adjusted for covariates, sex modified the association between water fluoride and snoring (*p* interaction = < 0.001) such that higher water fluoride concentrations were associated with lower odds of self-reported snoring among males (OR = 0.62, *p* = 0.03). Specifically, each IQR increase in water fluoride was associated with 0.62 times the odds of males reporting snoring *ever* compared to *never.* Water fluoride concentrations were not associated with odds of snoring among females (OR = 1.26, uncorrected *p* = .28).

There were also sex differences in the association between plasma fluoride and sleep apnea symptoms (*p* interaction = 0.09). Among males, higher plasma fluoride concentrations were associated with higher odds of reporting sleep apnea symptoms, although this did not reach the threshold for statistical significance (uncorrected *p* = 0.17). We did not observe any other interactions between fluoride and sex in relation to sleep outcomes. Plasma fluoride concentrations were not significantly associated with any of the other sleep outcome measures examined herein (uncorrected *ps:* 0.1–0.8; Additional file 2: Table S3).

### Sensitivity analysis

Cotinine-adjusted associations between plasma fluoride and sleep outcomes are presented in Additional file 2: Table S4. Median to 75th percentile serum cotinine concentrations ranged from 0.04 to 0.58 ng/mL, indicating that most participants had low recent nicotine exposure. The findings from survey-weighted covariate adjusted models did not change appreciably with the addition of serum cotinine as a covariate.

## Discussion

To our knowledge, this is the first published study to explore the relationship between fluoride exposure and sleep patterns in either humans or animals. We examined this relationship among a nationally representative sample of older adolescents living in the US, a country with ubiquitous fluoride exposure, and adjusted for factors that can affect fluoride exposure/metabolism and sleep patterns. We found that each 0.52 mg/L increase in household tap water fluoride concentration was associated with a 1.97 times higher likelihood of adolescents reporting having experienced symptoms suggestive of sleep apnea at least once per week. This remained significant after stringent corrections for multiple comparisons. Specifically, higher water fluoride concentrations were associated with higher odds of participants reporting *snorting, gasping or stopping breathing* while sleeping at night. This suggests that fluoride exposure at population-relevant levels may be a risk factor for sleep disturbances; however, additional studies are needed to explore this possibility, given the scarcity of data on this topic.

Our findings also showed that fluoride exposure may be associated with shifts in the sleep-wake cycle, as higher water fluoride concentrations were associated with later weekday bedtime and wake time, but not sleep duration. Specifically, for each 0.52 mg/L increase in adolescents’ water fluoride concentrations, they tended to report going to bed 24-min later and getting out of bed 26-min later. Additionally, there was some indication that adolescents with higher water fluoride concentrations may experience more frequent daytime sleepiness; however, future research is needed to explore this possibility.

The high accumulation of fluoride in pineal gland hydroxyapatite (among those chronically exposed) [9–11] points to a plausible mechanism by which fluoride may influence sleep patterns. In adults, pineal gland fluoride concentrations have been shown to strongly correlate with degree of pineal gland calcification [9, 11]. Interestingly, greater degree of pineal calcification among older adolescents and/or adults is associated with decreased melatonin production [28], lower REM sleep percentage, decreased total sleep time, poorer sleep efficiency [29], greater sleep disturbances and greater daytime tiredness [30]. While there are no existing human studies on fluoride exposure and melatonin production or sleep behaviors, findings from a doctoral dissertation demonstrated that gerbils fed a high fluoride diet had lower nighttime melatonin production than those fed a low fluoride diet. Moreover, their melatonin production was lower than normal for their developmental stage [31]. Interestingly, there is emerging evidence that melatonin may be an effective treatment for adult and pediatric sleep disturbances, including central and obstructive sleep apnea, which suggests that low melatonin production may play an etiological role in these disorders [32–34]. Therefore, it is possible that excess fluoride exposure may contribute to increased pineal gland calcification and subsequent decreases in nighttime melatonin production that contribute to sleep disturbances. Additional animal and prospective human studies are needed to explore this hypothesis.

There are also other mechanisms by which fluoride exposure could potentially contribute to sleep disturbances. For example, higher water fluoride concentrations are associated with an increased likelihood of hypothyroidism among adults [35, 36] and an increased incidence of diabetes among both adults [37] and children [38]. Both conditions are associated with an increased risk of obstructive sleep apnea in adulthood [39–41]. Likewise, in adults with Type 2 diabetes, lower melatonin production is positively associated with the presence and severity of sleep apnea symptoms [42, 43]. Additionally, prenatal and childhood fluoride exposure is associated with higher prevalence of Attention-deficit/hyperactivity disorder (ADHD) symptoms and diagnoses [44–46]. Prospective research has shown that individuals with childhood ADHD that persists into late adolescence/young adulthood tend to have poorer sleep quality by that time [47]. Thus, fluoride exposure could potentially increase the risk of sleep disturbances via interference with other endocrine or neurodevelopmental processes that influence sleep. Conversely, sleep disturbances may also contribute to ADHD symptoms [48, 49], as well as adversely affect endocrine health [50].

Intriguingly, we also found that each 0.52 mg/L increase in household tap water fluoride concentration was associated with a 38% reduction in the likelihood of male adolescents reporting snoring. Given that snoring is more prevalent among males [51], it is expected that reductions in snoring would also be more likely to occur among this population. Snoring tends to be most predominant during slow wave sleep [52]; thus, we speculate that our findings may point to a role of fluoride exposure in disrupting this deep sleep stage, thereby reducing opportunities for snoring. Consistently, youth with idiopathic central sleep apnea have been shown to experience reductions in slow-wave sleep [53], and water fluoride was positively associated with reported sleep apnea symptoms in our study. Alternatively, another possibility is that the gains in oral health from consumption of fluoridated water may protect against tonsillar infections that can contribute to snoring [54]. Future studies are needed to explore potential mechanisms by which fluoride exposure may reduce self-reported snoring.

Interestingly, while water fluoride concentrations were significantly associated with several measured sleep outcomes, plasma fluoride concentrations were not associated with any. Household tap water fluoride concentrations may serve as a proxy for long-term exposure (if place of residence does not change) and therefore, our findings may point to a role of childhood or early adolescent fluoride exposure in altering sleep regulation. However, since participants were enrolled during or after 2015, the year that the Public Health Service recommended lowering water fluoride concentrations from 0.7–1.2 mg/L to 0.7 mg/L, such effects would have occurred at higher water fluoride concentrations than those observed in this study [27]. Future prospective studies are needed to examine critical windows of vulnerability for potential effects of fluoride exposure on sleep, as well as whether very low water fluoride concentrations, such as those observed in this study, are a potential risk factor for sleep disturbances.

Additionally, it is important to consider that our results and those of others on adverse effects of fluoride exposure must be balanced with the long-standing evidence of the benefit of fluoride on oral health. In this manner, our work also contributes to development and refinement of policies on delivery of fluoride to the public.

## Limitations

There are several limitations in this study. First, sleep outcomes were measured via self- report which may be subject to biases including recall inaccuracies [55, 56]. Future studies employing actigraphy or polysomnography are needed to more objectively examine the association between fluoride exposure and sleep outcomes. Second, blood sample collection time was not standardized which can contribute to exposure misclassification and bias estimates toward the null. Third, while household tap water fluoride concentrations may serve as a proxy for long-term fluoride exposure, NHANES did not provide data on participants’ length of time at their current residence and thus we could not determine their duration of exposure to the water fluoride concentrations measured in this study. Fourth, participants were older adolescents who may be prone to sleep disruptions for various reasons, including playing video games, studying, working at jobs or having social influences, for example. Future studies should expand the age range to examine associations between fluoride exposure and sleep in both younger and older individuals, as well as to examine effects of cumulative fluoride exposure. Lastly, in cross-sectional studies, it is difficult to determine directionality of associations found; however, we would not expect reverse causality of the relationships observed in this study whereby sleep behaviors influence water fluoride concentrations. Still, the cross-sectional nature of this study does not allow for examination of critical windows of vulnerability for potential effects of fluoride exposure on sleep regulation which future prospective studies will be needed to address.

## Conclusions

Chronic low-level fluoride exposure may contribute to changes in sleep cycle regulation and sleep behaviors among older adolescents in the US. Additional prospective studies are warranted to examine effects of fluoride on sleep patterns and determine critical windows of vulnerability for potential effects.

## Supplementary information

**Additional file 1: Figure S1.** Participant selection flow chart

**Additional file 2: Table S1.** A Comparison of demographic characteristics between the study sample and all adolescents in NHANES 2015-2016. **Table S2.** Quasi-likelihood estimation of fluoride exposure with bedtime and wake time. **Table S3.** Associations between plasma fluoride concentrations and sleep outcomes. **Table S4.** Sensitivity analysis adjusting for serum cotinine in associations of plasma fluoride with sleep outcomes

## Data Availability

Data from the National Health and Nutrition Survey (2015–2016) is publicly available: [https://wwwn.cdc.gov/nchs/nhanes/ContinuousNhanes/Default.aspx?BeginYear=2015]. The datasets used and/or analyzed during the current study are available from the corresponding author on reasonable request.

## Abbreviations

ADHD: Attention-deficit/hyperactivity disorder
BMI: Body mass index
CAPI: Computer-Assisted Personal Interview
IQR: Interquartile range
LLOD: Lower limit of detection
NCHS: National Center for Health Statistics.
NHANES: National Health and Nutrition Examination Survey
SP: Study participant
US: United States
