# Supplementary material for: Fluoride exposure and sleep patterns among older adolescents in the United States: a cross-sectional study of NHANES 2015–2016
Source: Environ Health. 2019 Dec 9;18:106. doi: 10.1186/s12940-019-0546-7 (PMC6902325; doi:10.1186/s12940-019-0546-7)
Supplement: Supplementary file 1 — Additional file 1: Figure S1. Participant selection flow chart [file 12940_2019_546_MOESM1_ESM.docx]

n

**Participants in NHANES 2015-2016**

n = 9971

**Participants ages 16-19**

n = 608

**Plasma or water fluoride measurements**

n = 584

**Figure S1. Participant selection flow chart**

**Water fluoride sample**

n = 419

Participants with water fluoride data

n = 503

**Plasma fluoride sample**

n = 473

**Sample that met inclusion criteria**

n = 512

**Complete covariate data and at least one sleep outcome**

n = 515

**Exclude:**

Participants prescribed sleep medication (n = 3)

N=3

GFR =< 60

**Exclude:**

Participants who do not drink tap water

(n = 84)
