# Supplementary material for: Fluoride exposure and sleep patterns among older adolescents in the United States: a cross-sectional study of NHANES 2015–2016
Source: Environ Health. 2019 Dec 9;18:106. doi: 10.1186/s12940-019-0546-7 (PMC6902325; doi:10.1186/s12940-019-0546-7)
Supplement: Supplementary file 2 — Additional file 2: Table S1. A Comparison of demographic characteristics between the study sample and all adolescents in NHANES 2015-2016. Table S2. Quasi-likelihood estimation of fluoride exposure with bedtime and wake time. Table S3. Associations between plasma fluoride concentrations and sleep outcomes. Table S4. Sensitivity analysis adjusting for serum cotinine in associations of plasma fluoride with sleep outcomes [file 12940_2019_546_MOESM2_ESM.docx]

**Table S1. A Comparison of demographic characteristics between the study sample and all adolescents in NHANES 2015-2016**

| **Demographic characteristic** | | **Current study sample**  n = 512 | | **All adolescents**  **16-19 years**  N = 608 |
| --- | --- | --- | --- | --- |
| **Age (yrs.); Mean (SE)** | | 17.33 (0.05) | 17.38 (0.05) | |
| **Ratio of family income to poverty ^a^** | | 2.01 (0.07) | 2.01 (0.07) | |
| **Sex; N (%)**  Male  Female | | 253 (49.41)  259 (50.59) | 301 (49.51)  307 (50.49) | |
| **BMI categories ^b^; N (%):**  Underweight  Normal weight  Overweight  Obese | | 17 (3.35)  283 (55.82)  104 (20.51)  103 (20.32) | 19 (3.33)  316 (55.44)  117 (20.53)  118 (20.70) | |
| **Race/ethnicity; N (%)** |  | |  | |
| Mexican American  Other Hispanic  Non-Hispanic White  Non-Hispanic Black  Non-Hispanic Asian  Other Race-  (Including Multi-Racial) | 120 (23.44)  51 (9.96)  140 (27.34)  108 (21.09)  62 (12.11)  31 (6.05) | | 142 (23.36)  66 (10.86)  153 (25.16)  140 (20.0)  73 (12.01)  34 (5.59) | |

*Note.* ^a^ n=537 for all adolescents; ^b^ n = 507 for current study sample due to missing data on these variables

**Table S2. Quasi-likelihood estimation of fluoride exposure with bedtime and wake time**

|  |  | **Water fluoride** |  |  | **Plasma fluoride** |  |
| --- | --- | --- | --- | --- | --- | --- |
|  | **N** | **B (95% CI)** | **Uncorrected *p*** | **N** | **B (95% CI)** | **Uncorrected *p*** |
| **Bedtime** | 418 | 0.36 (0.12, 0.59) | 0.003 | 472 | -0.08 (- 0.20, 0.45) | 0.24 |
| **Wake time** | 418 | 0.43 (0.18, 0.69) | 0.001 | 472 | 0.09 (-0.06, 0.24) | 0.23 |

*Note.* Beta estimates reflect the change in outcome for each IQR (i.e. 0.52 mg/L for water fluoride and 0.19 µmol/L for plasma fluoride) increase in water or plasma fluoride. Regression analyses were adjusted for age, sex, race/ethnicity, body mass index, and ratio of family income to poverty. These analyses are unweighted.

**Table S3. Associations between plasma fluoride and sleep measures**

| **Outcomes** | **N** | **Estimates (95% CI)** | **Uncorrected *p*** | **Holm-Bonferroni corrected *p*** |
| --- | --- | --- | --- | --- |
| **Sleep duration**  Less than recommended  Recommended (ref)  More than recommended | 472 | 0.98 (0.79, 1.20)  ---  1.13 (0.93, 1.36) | 0.19^†^  0.83  ---  0.22 | 1.00  ---  1.00 |
| **Sleep apnea symptoms**  Never (ref)  At least once per week | 466 | ---  1.39 (0.87, 2.20) ^a^ | ---  0.17 | 1.00 |
| **Snoring**  Never (ref)  At least once per week | 454 | ---  0.91 (0.74, 1.12) | 0.38 | 1.00 |
| **Daytime sleepiness**  Never (ref)  Rarely  Sometimes  Often  Almost always | 473 | ---  1.17 (0.89, 1.55)  0.89 (0.71, 1.12)  0.95 (0.79, 1.13)  0.84 (0.58, 1.23) | 0.12^ǂ^  0.26  0.33  0.55  0.36 | ---  1.00  1.00  1.00  1.00 |
| **Trouble sleeping**  No (ref)  Yes | 473 | ---  1.11 (0.93, 1.31) | 0.25 | ---  1.00 |
| **Bedtime** | 472 | -0.08 (-0.19, 0.02) | 0.11 | 0.78 |
| **Wake time** | 472 | -0.01 (-0.11, 0.09) | 0.81 | 1.00 |

*Note*. All estimates are odds radios (ORs) except for sleep time and wake time which are unstandardized Beta estimates; ORs and Beta estimates reflect the change in outcome for each IQR (i.e. 0.19 µmol/L) increase in plasma fluoride. Regression analyses were adjusted for age, sex, race/ethnicity, body mass index, and ratio of family income to poverty. Sampling weights were applied to these regression analyses. †The p-value for a Type 3 Analysis of Effects with 2 degrees of freedom; ǂ The p-value for a Type 3 Analysis of Effects with 4 degrees of freedom; ^a.^ Odds ratio for association between plasma fluoride and sleep apnea symptoms among males; interaction between sex and plasma fluoride in predicting sleep apnea symptoms (B = - 1.74, *p* = 0.09)

**Table S4. Sensitivity analysis adjusting for serum cotinine in associations of plasma fluoride with sleep measures**

| **Outcomes** | **N** | **Estimates (95% CI)** | **Uncorrected**  ***p*** | **Holm-Bonferroni corrected *p*** |
| --- | --- | --- | --- | --- |
| **Sleep duration**  Less than recommended  Recommended (ref)  More than recommended | 466 | 0.96 (0.79, 1.16)  1.09 (0.91, 1.30) | 0.35^†^  0.64  0.37 | 1.00  1.00 |
| **Sleep apnea symptoms**  Never (ref)  At least once per week | 460 | 1.33 (0.81, 2.19) ^a^ | 0.26 | 1.00 |
| **Snoring**  Never (ref)  At least once per week | 448 | 0.90 (0.71, 1.14) | 0.40 | 0.80 |
| **Daytime sleepiness**  Never (ref)  Rarely  Sometimes  Often  Almost always | 467 | 1.15 (0.84, 1.56)  0.89 (0.71, 1.11)  0.92 (0.76, 1.12)  0.83 (0.57, 1.21) | 0.10^ǂ^  0.38  0.29  0.40  0.33 | 1.00  1.00  1.00  1.00 |
| **Trouble sleeping**  No (ref)  Yes | 467 | 1.12 (0.95, 1.33) | 0.19 | 0.94 |
| **Bedtime** | 466 | -0.10 (-0.21, 0.01) | 0.08 | 0.55 |
| **Wake time** | 466 | -0.03 (-0.13, 0.06) | 0.50 | 0.50 |

*Note*. All estimates are odds radios (ORs) except for sleep time and wake time which are unstandardized Beta estimates; ORs and Beta estimates reflect the change in outcome for each IQR (i.e. 0.19 μmol/L) increase in plasma fluoride; Regression analyses were adjusted for age, sex, race/ethnicity, body mass index, ratio of family income to poverty and serum cotinine. Median to 75^th^ percentile serum cotinine concentrations ranged from 0.04 to 0.58 ng/mL. Sampling weights were applied to these regression analyses. †The p-value for a Type 3 Analysis of Effects with 2 degrees of freedom; ǂ The p-value for a Type 3 Analysis of Effects with 4 degrees of freedom; ^a^ Odds ratio for association between plasma fluoride and sleep apnea symptoms among males; interaction between sex and plasma fluoride in predicting sleep apnea symptoms (B = - 1.57, *p* = 0.16)
